# The Psychology of Conspiracy Theories

**DOI:** 10.1177/0963721417718261

**Published:** 2017-12-07

**Authors:** Karen M. Douglas, Robbie M. Sutton, Aleksandra Cichocka

**Affiliations:** School of Psychology, University of Kent

**Keywords:** conspiracy theories, conspiracy belief, motives, needs

## Abstract

What psychological factors drive the popularity of *conspiracy theories*, which explain important events as secret plots by powerful and malevolent groups? What are the psychological consequences of adopting these theories? We review the current research and find that it answers the first of these questions more thoroughly than the second. Belief in conspiracy theories appears to be driven by motives that can be characterized as epistemic (understanding one’s environment), existential (being safe and in control of one’s environment), and social (maintaining a positive image of the self and the social group). However, little research has investigated the consequences of conspiracy belief, and to date, this research does not indicate that conspiracy belief fulfills people’s motivations. Instead, for many people, conspiracy belief may be more appealing than satisfying. Further research is needed to determine for whom, and under what conditions, conspiracy theories may satisfy key psychological motives.

Over a third of Americans believe that global warming is a hoax (Swift, 2013), and over half believe that Lee Harvey Oswald did not act alone in the assassination of John F. Kennedy (Jensen, 2013). These are examples of *conspiracy theories*—explanations for important events that involve secret plots by powerful and malevolent groups (e.g., Goertzel, 1994). In recent years, there has been growing interest in the psychological factors that drive the popularity of conspiracy theories, and in this article, we draw together and organize findings from this burgeoning research. This research suggests that people may be drawn to conspiracy theories when—compared with nonconspiracy explanations—they promise to satisfy important social psychological motives that can be characterized as epistemic (e.g., the desire for understanding, accuracy, and subjective certainty), existential (e.g., the desire for control and security), and social (e.g., the desire to maintain a positive image of the self or group). This taxonomy, derived from system-justification theory (Jost, Ledgerwood, & Hardin, 2008), serves as a useful heuristic to classify the motives associated with conspiracy belief. However, the comparatively scarce research examining the consequences of conspiracy theories does not indicate that they ultimately help people fulfill these motives.

## Epistemic Motives

Finding causal explanations for events is a core part of building up a stable, accurate, and internally consistent understanding of the world (Heider, 1958). Specific epistemic motives that causal explanations may serve include slaking curiosity when information is unavailable, reducing uncertainty and bewilderment when available information is conflicting, finding meaning when events seem random, and defending beliefs from disconfirmation. Relevant to these motives, conspiracy theories have attributes that set them apart from other types of causal explanation. Albeit to varying degrees, they are speculative in that they posit actions that are hidden from public scrutiny, complex in that they postulate the coordination of multiple actors, and resistant to falsification in that they postulate that conspirators use stealth and disinformation to cover up their actions—implying that people who try to debunk conspiracy theories may, themselves, be part of the conspiracy (Lewandowsky et al., 2015). A related property of conspiracy theories is that they can protect cherished beliefs (e.g., vaccination is harmful; climate change is not a serious concern) by casting overwhelmingly disconfirmatory evidence (e.g., scientific findings) as the product of a conspiracy (Lewandowsky, Oberauer, & Gignac, 2013).

In general, empirically warranted (vs. speculative), parsimonious (vs. complex), and falsifiable explanations are stronger according to normative standards of causal explanation (e.g., in science; see Grimes, 2016). However, conspiracy theories appear to provide broad, internally consistent explanations that allow people to preserve beliefs in the face of uncertainty and contradiction. In keeping with this analysis, research suggests that belief in conspiracy theories is stronger when the motivation to find patterns in the environment is experimentally heightened (Whitson & Galinsky, 2008). It is also stronger among people who habitually seek meaning and patterns in the environment, including believers in paranormal phenomena (e.g., Bruder, Haffke, Neave, Nouripanah, & Imhoff, 2013; but see Dieguez, Wagner-Egger, & Gauvrit, 2015). It also appears to be stronger when events are especially large in scale or significant and leave people dissatisfied with mundane, small-scale explanations (Leman & Cinnirella, 2013). Furthermore, the need for cognitive closure is associated with beliefs in salient conspiracy theories for events that lack clear official explanations (Marchlewska, Cichocka, & Kossowska, 2017). Also, research suggests that conspiracy belief is stronger when people experience distress as a result of feeling uncertain (van Prooijen & Jostmann, 2013).

Our analysis suggests that conspiracy theories may satisfy some epistemic motives at the expense of others—for example, by shielding beliefs from uncertainty while being less likely to be accurate. The epistemic drawbacks of conspiracy theories do not seem to be readily apparent to people who lack the ability or motivation to think critically and rationally. Conspiracy belief is correlated with lower levels of analytic thinking (Swami, Voracek, Stieger, Tran, & Furnham, 2014) and lower levels of education (Douglas, Sutton, Callan, Dawtry, & Harvey, 2016). It is also associated with the tendency to overestimate the likelihood of co-occurring events (Brotherton & French, 2014) and the tendency to perceive agency and intentionality where it does not exist (Douglas et al., 2016).

In light of their objective or normative limitations, how well do conspiracy theories satisfy the epistemic motives that draw people to them? Relatively little research has addressed this question, and it suggests that they may be more appealing than satisfying. On one hand, extreme and entrenched attitude positions are associated with conspiracy beliefs, suggesting that they may help people defend beliefs from disconfirmation (Uscinski, Klofstad, & Atkinson, 2016). In contrast, recent experiments indicate that presenting people with persuasive cases for conspiracy theories about vaccination (Jolley & Douglas, 2014a) and climate change (Jolley & Douglas, 2014b) increases their levels of uncertainty.

## Existential Motives

As well as their purely epistemic purposes, causal explanations serve the need for people to feel safe and secure in their environment and to exert control over the environment as autonomous individuals and as members of collectives (Tetlock, 2002). Several early theories of conspiracy belief suggested that people turn to conspiracy theories for compensatory satisfaction when these needs are threatened. For example, people who lack instrumental control may be afforded some compensatory sense of control by conspiracy theories, because they offer them the opportunity to reject official narratives and feel that they possess an alternative account (Goertzel, 1994). Conspiracy theories may promise to make people feel safer as a form of cheater detection, in which dangerous and untrustworthy individuals are recognized and the threat they posed is reduced or neutralized (Bost & Prunier, 2013).

Research supports this account of the motivation behind conspiracy belief. Studies have shown that people are likely to turn to conspiracy theories when they are anxious (Grzesiak-Feldman, 2013) and feel powerless (Abalakina-Paap, Stephan, Craig, & Gregory, 1999). Other research indicates that conspiracy belief is strongly related to lack of sociopolitical control or lack of psychological empowerment (Bruder et al., 2013). Experiments have shown that compared with baseline conditions, conspiracy belief is heightened when people feel unable to control outcomes and is reduced when their sense of control is affirmed (van Prooijen & Acker, 2015).

Unfortunately, research conducted thus far does not indicate that conspiracy belief effectively satisfies this motivation. On the contrary, experimental exposure to conspiracy theories appears to immediately suppress people’s sense of autonomy and control (Douglas & Leite, 2017; Jolley & Douglas, 2014a, 2014b). These same studies have also shown that it makes people less inclined to take actions that, in the long run, might boost their autonomy and control. Specifically, they are less inclined to commit to their organizations and to engage in mainstream political processes such as voting and party politics. Furthermore, exposure to conspiracy theories may subtly undermine people’s autonomy in another way. Douglas and Sutton (2008) showed that people were effectively persuaded by proconspiracy material but were not aware that they had been persuaded and falsely recalled that their preexposure beliefs were identical to their new beliefs. Since conspiracy theories suggest that important outcomes are in the hands of malevolent forces who possess and exercise powers beyond legitimate limits, it would not be surprising if further research suggests that their effect is often disempowering.

## Social Motives

Causal explanations, conspiracy explanations included, are also informed by various social motivations, including the desire to belong and to maintain a positive image of the self and the in-group. Scholars have suggested that conspiracy theories valorize the self and the in-group by allowing blame for negative outcomes to be attributed to others. Thus, they may help to uphold the image of the self and the in-group as competent and moral but as sabotaged by powerful and unscrupulous others. If this is the case, we can expect conspiracy theories to be particularly appealing to people who find the positive image of their self or in-group to be threatened (Cichocka, Marchlewska, & Golec de Zavala, 2016).

Research generally supports this expectation. Experimental results suggest that experiences of ostracism cause people to believe in superstitions and conspiracy theories, apparently as part of an effort to make sense of their experience (Graeupner & Coman, 2017). Members of groups who have objectively low (vs. high) status because of their ethnicity (Crocker, Luhtanen, Broadnax, & Blaine, 1999) or income (Uscinski & Parent, 2014) are more likely to endorse conspiracy theories. People on the losing (vs. winning) side of political processes also appear more likely to believe conspiracy theories (Uscinski & Parent, 2014). Conspiracy belief has also been linked to prejudice against powerful groups (Imhoff & Bruder, 2014) and those perceived as enemies (Kofta & Sedek, 2005).

These findings suggest that conspiracy theories may be recruited defensively, to relieve the self or in-group from a sense of culpability for their disadvantaged position. In keeping with this defensive motivation, conspiracy belief is associated with narcissism—an inflated view of oneself that requires external validation and is linked to paranoid ideation (Cichocka, Marchlewska, & Golec de Zavala, 2016). Conspiracy belief is also predicted by *collective* narcissism—a belief in the in-group’s greatness paired with a belief that other people do not appreciate it enough (Cichocka, Marchlewska, Golec de Zavala, & Olechowski, 2016). Groups who feel that they have been victimized are more likely to endorse conspiracy theories about powerful out-groups (Bilewicz, Winiewski, Kofta, & Wójcik, 2013).

Although people are clearly attracted to conspiracy theories when their social motivations are frustrated, it is not at all clear that adopting these theories is a fruitful way to fulfill these motivations. A feature of conspiracy theories is their negative, distrustful representation of other people and groups. Thus, it is plausible that they are not only a symptom but also a cause of the feelings of alienation and anomie—a feeling of personal unrest and lack of understanding of the social world—with which they are correlated (e.g., Abalakina-Paap et al., 1999). Experiments show that exposure to conspiracy theories decreases trust in governmental institutions, even if the conspiracy theories are unrelated to those institutions (Einstein & Glick, 2015). It also causes disenchantment with politicians and scientists (Jolley & Douglas, 2014a). So far, therefore, empirical research suggests that conspiracy theories serve to erode social capital and may, if anything, frustrate people’s need to see themselves as valuable members of morally decent collectives.

## Summary, Caveats, and Future Research

Research thus far has successfully articulated some of the motivations that, together with deficiencies in available information, cognitive ability, and motivation to think critically, may contribute to conspiracy belief. Although scholars have theorized about the consequences of conspiracy beliefs for their adherents and the community, relatively little empirical research has been done to explore them. Nevertheless, preliminary work suggests that despite the allure of conspiracy beliefs for people who have heightened epistemic, existential, and social motives, they may ultimately thwart those motives further. In this sense, conspiracy theories might be seen as an ironic or self-defeating manifestation of motivated social cognition. There are grounds to expect further research to corroborate this preliminary picture since, as we have seen, conspiracy theories have some attributes that do not lend themselves to the fulfillment of these motives—for example, they are generally speculative and contrarian, represent the public as ignorant and at the mercy of unaccountable powers, and impute highly antisocial and cynical motives to other individuals.

Nonetheless, there are also grounds to expect future research to show that conspiracy theories fulfill the needs of some people. The experimental research conducted thus far has sampled from populations (undergraduate students and survey panelists) that are not particularly disadvantaged or threatened and that generally do not endorse conspiracy theories. For these people, conspiracy theories are likely to be experienced as unsettling, destabilizing, and potentially alienating. However, these people are not whom scholars have had in mind when they have argued that conspiracy theories may sometimes be adaptive. They include groups and individuals who are already alienated from society and for whom conspiracy theories may offer some compensation. These include disempowered groups who may use conspiracy theories to subvert dominance hierarchies by formulating their own understanding of realities (Sapountzis & Condor, 2013) and by fostering solidarity and collective action (Adams, O’Brien, & Nelson, 2006). In these communities, and indeed in online communities in which conspiracy theories represent normative or even official positions (e.g., the 9/11 Truth movement), conspiracy belief may offer an important source of belonging and shared reality. Furthermore, history has repeatedly shown that corporate and political elites do conspire against public interests. Conspiracy theories play an important role in bringing their misdeeds into the light.

To conduct fair tests of the utility of conspiracy belief, controlled longitudinal and experimental investigations of disadvantaged and threatened populations are needed. In particular, future research needs to examine individuals whose psychological needs are chronically or experimentally threatened and determine whether conspiracy belief moves them closer to or further away from the fulfillment of these needs. In one such design, Jolley, Douglas, and Sutton (2017) exposed people to threats to the legitimacy of their social system. They found that the deleterious effects of these threats on satisfaction with the status quo were eliminated when participants were also exposed to conspiracy theories. Conspiracy theories therefore appeared to buffer people from the effects of threats to the status quo.

## Conclusion

We have reviewed the current literature on the psychological factors that appear to drive conspiracy belief. We conclude that conspiracy belief appears to stem to a large extent from epistemic, existential, and social motives. Research has yet to demonstrate that it effectively serves those motivations, and early indications are that it may often thwart them. It is possible, therefore, that conspiracy belief is a self-defeating form of motivated social cognition. However, important questions remain open, and more controlled research on the consequences of conspiracy beliefs is needed, particularly on the vulnerable and disadvantaged populations that have been identified as most likely to benefit from them. We hope that this review will serve as an organizing schema for future research on the psychology of conspiracy belief.
